# Flavanoids induce expression of the suppressor of cytokine signalling 3 (*SOCS3*) gene and suppress IL-6-activated signal transducer and activator of transcription 3 (STAT3) activation in vascular endothelial cells

**DOI:** 10.1042/BJ20130481

**Published:** 2013-08-09

**Authors:** Jolanta Wiejak, Julia Dunlop, Simon P. Mackay, Stephen J. Yarwood

**Affiliations:** *Institute of Molecular, Cell and Systems Biology, College of Medical, Veterinary and Life Sciences, University of Glasgow, Glasgow G12 8QQ, Scotland, U.K.; †Strathclyde Institute of Pharmacy and Biomedical Sciences, 161 Cathedral Street, University of Strathclyde, Glasgow G4 0RE, Scotland, U.K.

**Keywords:** flavanoid, gene expression, interleukin-6 (IL-6), Janus kinase/signal transducer and activator of transcription (JAK/STAT), suppressor of cytokine signalling 3 (SOCS3), ACC, acetyl-CoA carboxylase, AMPK, AMP-activated protein kinase, CK2, casein kinase 2, DMEM, Dulbecco’s modified Eagle’s medium, ERK, extracellular-signal-regulated kinase, HEK, human embryonic kidney, HUVEC, human umbilical vein endothelial cell, IL, interleukin, IL-6R, IL-6 receptor, JAK, Janus kinase, MAPK, mitogen-activated protein kinase, MCP-1, monocyte chemotactic protein-1, MEK, MAPK/ERK kinase, PDE, phosphodiesterase, PKC, protein kinase C, RT, reverse transcription, SH, Src homology, SOCS, suppressor of cytokine signalling, STAT3, signal transducer and activator of transcription 3, TNFα, tumour necrosis factor α, VASP, vasodilator-stimulated phosphoprotein, VEC, vascular endothelial cell

## Abstract

The atherogenic cytokine IL-6 (interleukin-6) induces pro-inflammatory gene expression in VECs (vascular endothelial cells) by activating the JAK (Janus kinase)/STAT3 (signal transducer and activator of transcription 3) signalling pathway, which is normally down-regulated by the STAT3-dependent induction of the E3 ubiquitin ligase component SOCS3 (suppressor of cytokine signalling 3). Novel treatments based on the regulation of SOCS3 protein levels could therefore have value in the treatment of diseases with an inflammatory component, such as atherosclerosis. To this end we carried out a screen of 1031 existing medicinal compounds to identify inducers of *SOCS3* gene expression and identified the flavanoids naringenin and flavone as effective inducers of SOCS3 protein, mRNA and promoter activity. This was in contrast with the action of traditional JAK/STAT3 inhibitors and the polyphenol resveratrol, which effectively suppress *SOCS3* gene expression. Both naringenin and flavone also effectively suppressed IL-6-stimulated phosphorylation of STAT3 (Tyr^705^) which led to suppression of IL-6-induction of the atherogenic STAT3 target gene *MCP1* (monocyte chemotactic protein-1), suggesting that their ability to induce *SOCS3* gene expression is STAT3-independent. Supporting this idea was the observation that the general kinase inhibitor compound C inhibits flavone- and cAMP-dependent, but not JAK-dependent, SOCS3 induction in VECs. Indeed, the ability of flavanoids to induce SOCS3 expression requires activation of the ERK (extracellular-signal-regulated kinase)-dependent transcription factor SP3, and not STAT3. In the present paper we therefore describe novel molecular actions of flavanoids, which control *SOCS3* gene induction and suppression of STAT3 signalling in VECs. These mechanisms could potentially be exploited to develop novel anti-atherogenic therapies.

## INTRODUCTION

Unchecked chronic inflammation is now thought to be responsible for many cancers and deadly cardiovascular diseases, including atherosclerosis. Chronic inflammation is associated with increased inflammatory activity associated with increased levels of pro-inflammatory cytokines in the circulation, including IL-1 (IL is interleukin), IL-6 and TNFα (tumour necrosis factor α) [1,2]. IL-6, along with TNFα and IL-1, is known to play a key role in B-cell maturation and T-cell differentiation, as well as driving acute inflammatory responses. However, sustained IL-6 production is involved in chronic low-level inflammation associated with many diseases, including obesity and insulin resistance, inflammatory bowel diseases, rheumatoid arthritis, sepsis, several forms of cancer and atherosclerosis [3,4]. Indeed, IL-6 has been detected in atherosclerotic plaques [1], and prolonged elevation of IL-6 is strongly associated with increased levels of cholesterol and hypertension [5,6]. Moreover, high IL-6 levels in elderly patients are associated with a 2-fold increase in both cardiovascular and other causes of mortality [7]. IL-6 affects VECs (vascular endothelial cells) by triggering counter-productive angiogenesis, through VEGF (vascular endothelial growth factor) production, and increasing the secretion of chemokines, such as MCP-1 (monocyte chemotactic protein-1), which recruit monocytes to the inflamed endothelium [1]. Signalling by IL-6 in VECs occurs through the IL-6R (IL-6 receptor) complex, composed of an IL-6-binding α chain (IL-6Rα) and gp130, which interacts with IL-6Rα [8]. It is IL-6R ‘*trans*-signalling’ [9], where IL-6 binds to soluble forms of IL6-Rα (sIL6-Rα) and triggering hyperactivation of gp130, that is thought to underlie the pro-inflammatory actions of IL-6 in a variety of diseases, including atherosclerosis [3]. Binding of the IL-6/sIL-6R complex to gp130 leads to receptor clustering and activation of the JAK (Janus kinase)/STAT3 (signal transducer and activator of transcription 3) signalling pathway and induction of pro-inflammatory IL-6-responsive genes, including MCP-1 and ICAM1 (intercellular adhesion molecule 1) [10].

Clearly, regulation of pro-inflammatory signalling is vital to prevent runaway inflammation, since progression of many diseases is associated with enhanced STAT3 activity. One mechanism for down-regulating JAK/STAT signalling is via the SOCS (suppressor of cytokine signalling) family of proteins [11], which are often induced directly by the same JAK/STAT pathway they inhibit, forming a classical negative-feedback loop [12]. For example, SOCS3 binds to JAK-phosphorylated receptors, via the SOCS3 SH (Src homology) domain (SH2), thereby inhibiting JAK/STAT3 signalling, and targeting SH2-bound proteins for proteasomal degradation [13]. Consistent with its role as a negative regulator of inflammatory signalling, SOCS3 expression is increased at sites of acute and chronic inflammation [14], and IL-6 has been reported to promote acute and chronic inflammatory disease in the absence of SOCS3 [15]. Moreover, conditional deletion of the *Socs3* gene in haemopoietic and endothelial cells of transgenic mice results in death caused by severe inflammatory lesions in the peritoneal and pleural cavities [16], illustrating its important protective role. Cell-permeable forms of recombinant SOCS3 have also been used to effectively suppress pathogen-induced acute inflammation by reducing the production of inflammatory cytokines, attenuating liver apoptosis and limiting haemorrhagic necrosis [17]. Clearly novel treatments based on the regulation of SOCS3 levels in cells could have value in the treatment of diseases such as atherosclerosis where there is hyperactivation of JAK/STAT3 signalling. To this end, we have identified the heterocyclic small molecules naringenin and flavone as small molecules that display the novel combined actions of IL-6-promoted STAT3 inhibitor and SOCS3-inducer in VECs. This is in contrast with the structurally related molecule resveratrol and other ‘traditional’ JAK inhibitors, which inhibit both IL-6-promoted STAT3 activation and SOCS3 induction. We suggest that by understanding the anti-inflammatory signalling mechanisms of small molecules such as naringenin and flavone, this may pave the way to the development of novel therapies based on the suppression of pro-inflammatory cytokine signalling.

## EXPERIMENTAL

### Materials

ECL reagents and secondary antibodies (horseradish peroxidase-conjugated anti-rabbit-IgG and horseradish peroxidase-conjugated anti-mouse-IgG) were bought from GE Healthcare. HUVECs (human umbilical vein endothelial cells) and endothelial cell growth medium 2 were obtained from PromoCell. Dulbecco's PBS was from Sigma–Aldrich. Forskolin, rolipram, PMA, compound C and MG132 were obtained from Merck. 5,7-dihydroxy-2-(4-hydroxyphenyl) chroman-4-one (naringenin) and 5-[(E)-2-(4-hydroxyphenyl)-vinyl]-1,3-benzenediol (*trans*-resveratrol) were from Sigma–Aldrich and Indofine Chemical Company. All other chemicals were purchased from Sigma–Aldrich, apart from 5-[(Z)-2-(4-hydroxyphenyl)-vinyl]-1,3-benzenediol (*cis*-resveratrol; Caymen Chemical Company), 2-phenyl-1H-quinolin-4-one (Synchem) and 3-phenyl-4*H*-chromen-4-one (isoflavone; Maybridge). The NINDS-II [NINDS (National Institute for Neurological Disorders and Stroke) Custom Collection II] was obtained from MRCT (Medical Research Council Technology).

### Plasmids

The minimal mouse *Socs3* promoter construct (pGL3-SOCS3-107Luc) was a gift from Professor J.G. Bode (Heinrich-Heine University, Dusseldorf, Germany) with permission from Professor Shlomo Melmed (Cedars-Sinai Medical Center, West Hollywood, CA, U.S.A.). This plasmid contains the promoter region −107 to +929 of the murine *Socs3* gene fused to the coding region of firefly luciferase as described previously [18]. PGL3-SOCS3-107Luc constructs mutated to disrupt the putative SP3, distal and proximal STAT-binding regions (dSTAT and pSTAT respectively), as described previously [19], were also obtained from Professor J.G. Bode. The QuikChange® Site-Directed Mutatgenesis kit (Agilent) was used to introduce mutations into vectors pGL3-SOCS3-107Luc, pGL3-SOCS3-107-pSTAT, pGL3-SOCS3-107SP3 and pGL3-SOCS3-107-pSTAT-SP3, using primers 5′-GCCTTTCAGTGCAGAGTAAAGCTTAAACATTACAAGAAGACCGGCCGGGC-3′ (forward) and 5′-GCCCGGCCGGTCTTCTTGTAATGTTTAAGCTTTACTCTGCACTGAAAGGC-3′ (reverse), to disrupt the putative AP1 site (G^−105^TGACTAA^−98^ to A^−105^AGCTTAA^−98^). Mutations were also introduced into vectors pGL3-SOCS3-107Luc, pGL3-SOCS3-107-pSTAT, pGL3SOCS3-107-SP3 and pGL3-SOCS3-107-pSTAT-SP3, using primers 5′-GCCTTTCAGTGCAGAGTAAAGCTTAAACATCCCAGGAAGACCGGCCGGGC-3′ (forward) and 5′-GCCCGGCCGGTCTTCCTGGGATGTTTAAGCTTTACTCTGCACTGAAAGGC-3′ (reverse), to disrupt both the putative AP1-binding site (G^−105^TGACTAA^−98^ to A^−105^AGCTTAA^−98^) together with the putative dSTAT site (T^−95^TACAAGAA^−87^ to T^−95^CCCAGGAA^−87^). The SP3-Luc (pAldGCB^4^luc; [19]) reporter construct was a gift from Professor Gerald Thiel (University of Saarland, Homberg, Germany), the STAT reporter construct was from Dr Timothy Palmer (University of Glasgow, Glasgow, Scotland, U.K.) and the AP1 reporter was from Professor Walter Kolch (University College Dublin, Dublin, Ireland).

### Cell culture and transfections

COS-1 and HEK (human embryonic kidney)-293 cells were grown in 75 cm^2^ tissue culture flasks in DMEM (Dulbecco's modified Eagle's medium; Sigma–Aldrich) supplemented with 10% (v/v) FBS (Sigma–Aldrich), 2 mM glutamine and 2% (v/v) penicillin/streptomycin (Sigma–Aldrich) at 37°C in a humidified 5% (v/v) CO_2_ atmosphere. HUVECs were grown in human endothelial cell growth medium 2 (PromoCell) at 37°C in a humidified 5% (v/v) CO_2_ atmosphere.

### Library screening

A 1.7 kbp fragment of the human *SOCS3* promoter cloned into pGL3-Basic (hSOCS3-1.7kbp) was provided by Dr Jason Mathews (University of Toronto, Toronto, ON, Canada) [20]. A minimal promoter truncate was then generated with the QuikChange® II Site-Directed Mutagenesis kit (Agilent) using this promoter fragment as an initial template. The primers used were hSOCS3-1.1kbp (forward, 5′-GCCGAGGCTGGGTAGCCCCTGCTCGCGGCC-3′, and reverse, 5′- GGCCGCGAGCAGGGGCTACCCAGCCTCGGC-3′). The resulting minimal promoter fragment was then excised from pGL3-Basic and subcloned into the multiple-cloning site of the pGL4-Basic vector (Promega) to generate the pGL4-hSOCS3-1.1 vector. For the NINDS-II library screen, COS-1 cells were seeded on to 96-well microtitre plates and then grown to approximately 80–90% confluence. Cells were then transfected with pGL4-hSOCS3-1.1 plus a *Renilla* luciferase reporter construct (pGL4.74) using DOTAP transfection agent (Roche), according to the manufacturer's instructions. The next day the medium was replaced with 200 μl of DMEM plus 10% (v/v) FBS containing 100 μM of individual compounds from the NINDS-II (MicroSource Discovery Systems) library. After overnight incubation the medium containing the compounds was removed and the cells washed with PBS. Cells were then lysed with passive lysis buffer (Promega) for 20 min with rocking at room temperature (18°C). Cell lysates were then collected and 20 μl samples were assayed in triplicate for luciferase activity using the Promega Dual Luciferase Reporter Assay System according to the manufacturer’s protocols. Luciferase activities were measured using a BMG Labtech luminometer.

### Immunoblotting

Cell lysates were prepared in sample buffer [50 mM Tris/HCl (pH 6.8), 2% (w/v) SDS, 10% (v/v) glycerol, 1% (v/v) 2-mercaptoethanol, 12.5 mM EDTA, 0.02% Bromophenol Blue and 100 mM DTT]. Protein samples were then separated by SDS/PAGE (10% gels), transferred on to nitrocellulose, blocked for 1 h at room temperature in 5% (w/v) BSA, and then immunoblotted with antibodies specific for SOCS3 (Santa Cruz Biotechnology), phospho-STAT3 (Tyr^705^), total STAT3 protein, phospho-ACC (acetyl-CoA carboxylase) (Ser^79^), total VASP (vasodilator-stimulated phosphoprotein), phospho-VASP (Ser^157^) or phospho-ERK1/2 (extracellular-signal-regulated kinase 1/2) (Thr^202^/Tyr^204^) and total ERK1/2 (New England Biolabs). Immunoblots were developed using ECL chemiluminescence (GE Healthcare).

### RT (reverse transcription)–PCR

Total RNA was isolated from HUVECs using an RNeasy Mini Kit (Qiagen) according to the manufacturer's instructions. RT–PCRs were carried out in a 25 μl reaction mixture containing 5–10 ng of RNA, 0.4 mM dNTPs and 0.6 μM of each primer using the OneStep RT-PCR Kit (Qiagen). The primer sequences used were: hSOCS3 (human SOCS3; forward, 5′-CACATGGCACAAGCACAAGA-3′, and reverse, 5′-AAGTGTCCCCTGTTTGGAGG-3′), hActin (human actin; forward, 5′-CTGGCACCCAGCACAATG-3′, and reverse, 5′-GCCGATCCACACGGAGTACT-3′) and hMCP-1 (human MCP-1; forward, 5′-GCTCAGCCAGATGCAATCAA-3′, and reverse, 5′-GGAGTTTGGGTTTGCTTGTC-3′). Reactions were carried out in a thermocycler with the following settings: 30 min at 50°C, 15 min at 95°C, 30 cycles of 30 s at 94°C, 30 s at 50°C and 1 min at 72°C, followed by 10 min at 72°C. PCR products were visualised on 2% (w/v) agarose gels following ethidium bromide staining.

### cAMP assay

HUVECs were incubated with pharmacological stimuli in 30 mm dishes for 5 h [37°C, 5% (v/v) CO_2_], washed with PBS (1×1 ml) and then incubated with 300 μl/dish of sample diluent from the DetectX High Sensitivity Direct Cyclic AMP Chemiluminescent Immunoassay kit from Arbor Assays. Cells were incubated with occasional mixing for a further 10 min at room temperature, scraped into Eppendorf tubes and then centrifuged at 1000 ***g*** for 15 min at 4°C. Supernatants were transferred to fresh tubes and frozen at −70°C. Detection of cAMP levels in standards and experimental samples was performed according to the manufacturer's protocols and chemiluminescent signals were captured using a BMG Labtech luminometer set to a 0.1 s read time per well of a 96-well plate.

### Statistics

Data were analysed using one-way ANOVA with a Tukey–Kramer post-test.

## RESULTS AND DISCUSSION

### Flavanoids induce *SOCS3* gene expression independently of JAK and STAT3 activation

**Figure 1:**
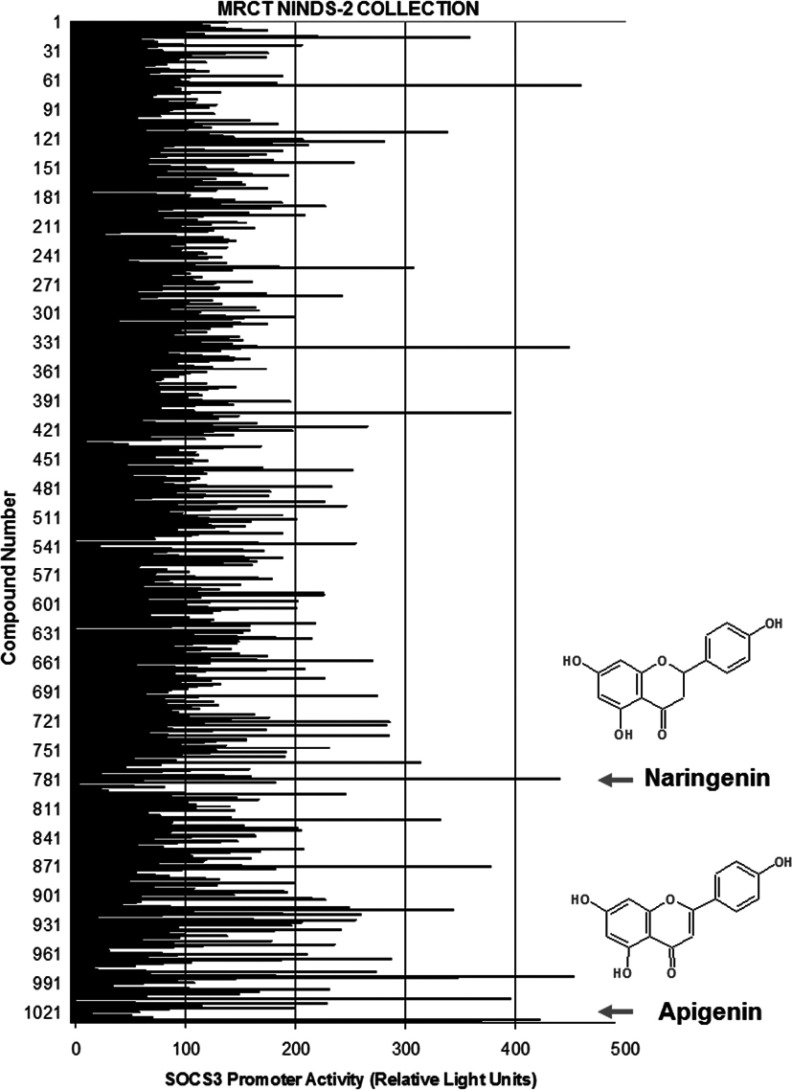
A high-throughput screen of medicinal compounds identifies naringenin as a potent inducer of *SOCS3* gene expression Luciferase activity data [RLU (relative light units)] derived from a screen of a human *SOCS3* minimal promoter/luciferase reporter construct transfected into COS-1 cells and screened with 1031 compounds from the 1040 compound NINDS-II compound collection as described in the Materials and methods section. The level of induction of *SOCS3* transcriptional activity induced by narigenin and apigenin treatment is shown with arrows, together with their relative structures.

In order to understand the molecular control of *SOCS3* gene expression we carried out a high-throughput screen of small molecules capable of inducing the expression of the minimal human *SOCS3* gene promoter [21] in a COS-1 cell-based dual-luciferase reporter assay. For the screen we used the NINDS-II medicinal compound library that was originally compiled by MicroSource Discovery Systems which we obtained from MRCT. The library consists of 1040 U.S.A. FDA (Food and Drug Administration)-approved compounds of known medicinal benefit, all at 100 μM. *SOCS3* promoter-expressing COS-1 cells were stimulated for 16 h with each compound and then luciferase activities were determined using a luminometer. The details of individual compounds and their respective abilities to regulate *SOCS3* promoter activity are detailed in the Supplementary Data File (at http://www.biochemj.org/bj/454/bj4540283add.htm). Of particular note from the screen was the observation that two structurally related flavanoid compounds, 5,7-dihydroxy-2-(4-hydroxyphenyl) chroman-4-one (naringenin) and 5,7-dihydroxy-2-(4-hydroxyphenyl)-4H-chromen-4-one (apigenin), both produced a robust (approximately 4-fold) induction of *SOCS3* promoter activity (Figure 1). Intriguingly, dietary naringenin is normally considered to have a positive bioactive effect on human health as a free radical scavenging antioxidant, an anti-inflammatory agent, an inducer of carbohydrate metabolism and immune system modulator [22]. Moreover, naringenin treatment has been shown to correct dyslipidaemia, hyperinsulinaemia and obesity, and attenuate atherosclerosis in mouse models of cardiovascular disease [23]. With this in mind, and as a first step to identifying potential sites for future pharmaceutical intervention to treat cardiovascular disease, we sought to investigate the molecular basis underlying the ability of naringenin to induce *SOCS3* gene activity in HUVECs, a cell model that is used routinely to study vascular endothelial cell function and pathology.

**Figure 2:**
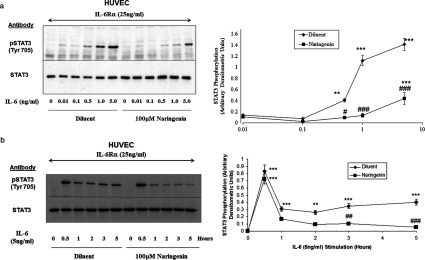
Naringenin inhibits cytokine-induced phosphorylation of STAT3 on Tyr^705^ (**a**) HUVECs were stimulated for 5 h with IL-6Rα (25 ng/ml) plus the indicated concentrations of IL-6, in the presence or absence of 100 μM naringenin. Cell extracts were then prepared and immunoblotted with anti-STAT3 and anti-phospho-STAT3 (Tyr^705^) antibodies as indicated. Densitometric values from three independent experiments are expressed as a graph in the right-hand panel (means±S.E.M.; *n*=3). Significant increases in STAT3 tyrosine phosphorylation, relative to cells stimulated with diluent alone are indicated (**P<0.01 and ***P<0.001 respectively), as are significant decreases in STAT3 tyrosine phosphorylation in IL-6 plus naringenin-treated cells relative to cells treated with naringenin alone (#*P*<0.05 and ###*P*<0.001 respectively). (**b**) HUVECs were stimulated for the indicated times with IL-6 (5 ng/ml) plus IL-6Rα (25 ng/ml) in the presence or absence of 100 μM naringenin. Tyrosine phosphorylation of STAT3 was determined by immunoblotting cell extracts with anti-STAT3 and anti-phospho-STAT3 (Tyr^705^) antibodies. Densitometric values from three independent experiments are expressed as a graph in the right-hand panel (means±S.E.M.; *n*=3). Significant increases in STAT3 tyrosine phosphorylation relative to cells stimulated with diluent alone are indicated (**P<0.01 and ***P<0.001 respectively), as are significant decreases in STAT3 tyrosine phosphorylation in IL-6 plus naringenin-treated cells relative to cells treated with naringenin alone (##*P*<0.01 and ###*P*<0.001 respectively).

We first examined the effects of naringenin on IL-6-regulated STAT3 activation, since STAT3 is a principle regulator of *SOCS3* expression in HUVECs [24]. We found that a 5 h treatment with 100 μM naringenin was able to significantly suppress the ability of IL-6 (5 ng/ml) to promote the activation-dependent Tyr^705^ phosphorylation of STAT3 in HUVECs [28.6±3.1% reduction (*n*=3, *P*<0.001); Figure 2a]. A time-course of IL-6-promoted STAT3 Tyr^705^ phosphorylation demonstrated that naringenin began to exert a significant inhibitory effect after approximately 3 h stimulation (Figure 2b). Another potential mechanism for inhibition of STAT3 activation is through inhibition of ERK-dependent STAT3 phosphorylation on Ser^727^, which is required for full STAT3 transcriptional activity [25]. However, Western blotting with phospho-specific antibodies revealed that incubation with naringenin did not affect the ability of IL-6 to activate ERK or promote STAT3 phosphorylation on Ser^727^ (Supplementary Figure S1 at http://www.biochemj.org/bj/454/bj4540283add.htm). From these results it is clear that 100 μM naringenin effectively inhibits tyrosine phosphorylation of STAT3 and it is therefore unlikely that the effects of naringenin on *SOCS3* induction are related to activation of STAT3 signalling. Indeed, results suggest that the ability of naringenin to inhibit tyrosine phosphorylation of STAT3, and yet induce *SOCS3* expression, may occur through distinct signalling mechanisms.

**Figure 3:**
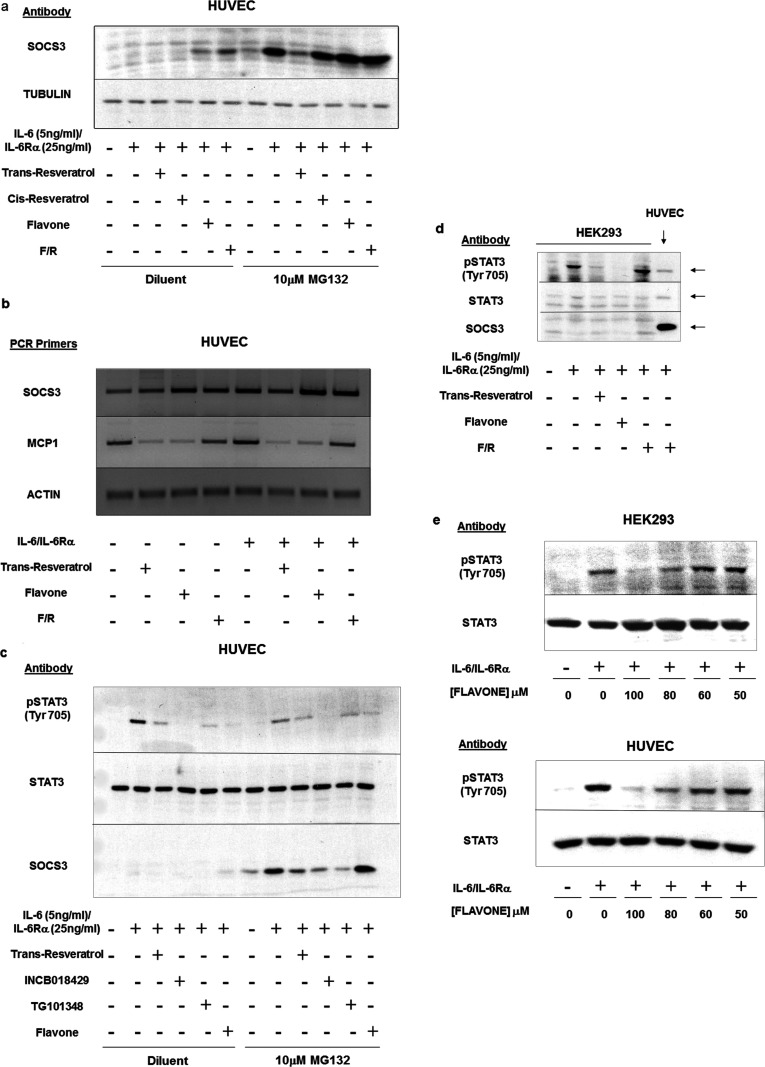
Naringenin and flavone, but not resveratrol, induce SOCS3 protein and mRNA expression (**a**) HUVECS were pre-incubated for 30 min in the presence or absence of MG132 (10 μM), to stabilize SOCS3 protein expression, and then for a further 5 h with IL-6 (5 ng/ml)/IL-6Rα (25 ng/ml) plus or minus the indicated chemical probes (100 μM). Cell extracts were prepared and immunoblotted with anti-SOCS3 or tubulin antibodies. (**b**) HUVECs were stimulated for 5 h in the presence or absence of IL-6 (5 ng/ml) plus IL-6Rα (25 ng/ml), forskolin/rolipram (F/R), 100 μM *trans*-resveratrol or 100 μM flavone. Cellular mRNA was then extracted and subjected to RT–PCR with specific primers to either human SOCS3, MCP-1 or actin, as described in the Materials and methods section. Results are representative of three separate experiments. (**c**) HUVECs were incubated in the presence or absence of MG132, IL-6 (5 ng/ml)/IL-6Rα (25 ng/ml), flavone, *trans*-resveratrol or the JAK inhibitors (10 μM) INCB018429 or TG101348. Cell extracts were probed with anti-SOCS3, anti-total-STAT3 or anti-phospho-STAT3 antibodies as indicated. (**d**) HEK-293 cells were stimulated with IL-6 (5 ng/ml) plus IL-6Rα (25 ng/ml) in the presence or absence of the indicated treatments. Cell extracts were then immuoblotted with anti-SOCS3, anti-STAT3 and anti-phospho-STAT3 antibodies. HUVEC extracts from cells that had been stimulated with F/R for 5 h were used as a positive control for immunoblots. (**e**) HEK-293 cells (top panel) and HUVECs (bottom panel) were incubated for 5 h with IL-6 (5 ng/ml) plus IL-6Rα (25 ng/ml) in the presence or absence of the indicated concentrations of flavone. Cell extracts were then prepared and immunoblotted with anti-STAT3 and anti-phospho-STAT3 antibodies as indicated.

**Figure 4:**
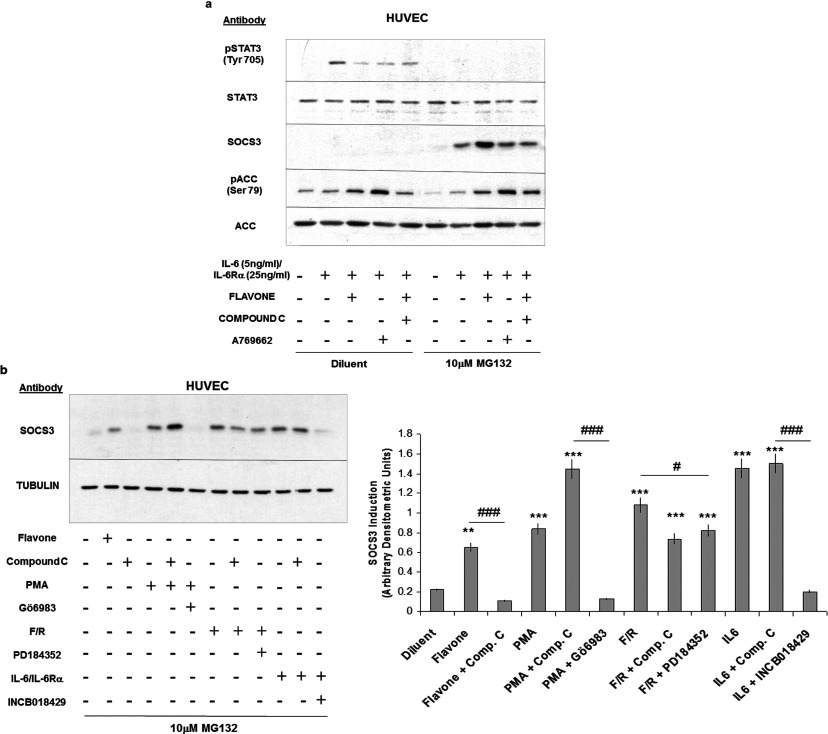
Compound C inhibits flavone- and cAMP-stimulated SOCS3 induction (**a**) HUVECs were pre-incubated for 30 min with either 100 μM flavone, 10 μM compound C or 10 μM of the AMPK activator A769662 and then stimulated for 5 h in the presence or absence of IL-6 (5 ng/ml)/IL-6Rα (25 ng/ml) plus MG132 (10 μM) as indicated. Cell extracts were then prepared and immunoblotted with the antibodies indicated. (**b**) HUVECs were incubated with the indicated combinations of pharmacological agents in the presence of 10 μM MG132 to stabilize cellular SOCS3 levels. Cell extracts were then immunoblotted with anti-SOCS3 and anti-tubulin antibodies, as indicated in the top panel, and densitometric values (means±S.E.M.) from immunoblots are displayed as a histogram in the right-hand panel. Significant increases in SOCS3 induction relative to cells stimulated with diluent alone are indicated (**P<0.01 and ***P<0.001 respectively), as are significant decreases in SOCS3 induction in cells treated with compound C, Gö6983, PD184352 and INCB018429 respectively, relative to cells treated with flavone, PMA, forskolin and rolipram (F/R) and IL-6 respectively (#*P*<0.05 and ###*P*<0.001).

To test this idea further, we examined the abilities of a range of structurally related flavanoids to both induce *SOCS3* promoter activity and inhibit STAT3 Tyr^705^ phosphorylation (Table 1). These results clearly demonstrated a lack of correlation between the ability of flavanoids to induce *SOCS3* promoter activity and inhibit STAT3 tyrosine phosphorylation (Table 1). For example, the level of suppression of IL-6-promoted STAT3 activation exhibited by 100 μM naringenin was comparable with that exerted by the naturally occurring STAT3 inhibitor 100 μM *trans*-resveratrol [26]; however, of the two, naringenin was the most effective at inducing *SOCS3* promoter activity (Table 1). Moreover, flavone, which represents the de-hydroxylated core-structure of naringenin, was found to be a much more effective inhibitor of STAT3 Tyr^705^ phosphorylation than naringenin, yet induced *SOCS3* promoter activity to similar level as naringenin (Table 1). To examine these effects further we investigated the ability of 100 μM flavone and 100 μM resveratrol to induce the expression of SOCS3 protein in HUVECs (Figure 3a). In this case cells were also incubated in the presence or absence of the proteasome inhibitor MG132 (Figure 3a), which prevents the proteolytic degradation of newly synthesized SOCS3 by the ubiquitin–proteasome system [10]. We also examined the effects of 100 μM flavone and 100 μM resveratrol treatment on the levels of *SOCS3* mRNA and mRNA levels of a STAT3-target gene, the atherogenic chemokine *MCP1*, in HUVECs (Figure 3b). *Trans*-resveratrol proved to be an effective inhibitor of IL-6-promoted SOCS3 protein and mRNA expression in HUVECs (Figures 3a and 3b), exerting similar inhibitory effects to two commercially available JAK inhibitors, INCB018429 and TG101348, which strongly suppress SOCS3 induction by IL-6 (Figure 3c). In contrast, flavone was found to be an effective inducer of SOCS3 protein and mRNA levels in HUVECs, inducing levels comparable with stimulation with a combination of the adenylate cyclase activator forskolin and the cAMP-specific PDE (phosphodiesterase) 4 inhibitor rolipram (F/R; Figure 4), and yet suppressed mRNA levels of *MCP1* to levels comparable with those achieved following *trans*-resveratrol treatment (Figure 3b). This suggests that flavone inhibits STAT3 activation independently of SOCS3 induction. Indeed, this idea is supported by the observation that flavone can inhibit STAT3 Tyr^705^ phosphorylation in HEK-293 cells, which lack an inducible *SOCS3* gene (Figure 3d). In these cells IL-6 was found to induce phosphorylation of STAT3 on Tyr^705^ and this was effectively inhibited by both *trans*-resveratrol and flavone, despite the lack of *SOCS3* expression (Figure 3d). Indeed, flavone was effective at inhibiting STAT3 tyrosine phosphorylation over the same range of concentrations in both SOCS3-negative HEK-293 cells and SOCS3-positive HUVECs (Figure 3e). Together these results demonstrate that the flavanoids naringenin and flavone suppress cytokine-induced STAT3 activation through SOCS3-independent mechanisms and, conversely, induce *SOCS3* gene activity through JAK/STAT3-independent mechanisms.

**Table 1:** Naringenin and its heterocyclic core, flavone, display the combined actions of STAT3 inhibitor and inducer of *SOCS3* promoter activity HUVECs were stimulated for 5 h with IL-6 (5 ng/ml) plus IL-6Rα (25 ng/ml) in the presence or absence of 100 μM of the compounds indicated. Cell lysates were then prepared and immunoblotted with anti-STAT3 and anti-phospho-STAT3 (Tyr^705^) antibodies. Immunoblots from three separate experiments were quantified and densitometric values (means±S.E.M.) are shown in the third column. Representative blots from immunoblot experiments are given in Supplementary Figure S2 (at http://www.biochemj.org/bj/454/bj4540283add.htm). Significant differences in the ability of chemical probes to inhibit IL-6-promoted STAT3 phosphorylation are indicated [*P<0.05 and ***P<0.001 (*n*=3)]. Alternatively, COS-1 cells were transfected with a *SOCS3* minimal promoter luciferase reporter construct, as described in the Materials and methods section, and the ability of chemical probes to induce promoter activity is indicated in the fourth column as the mean percentage change in relative light units±S.E.M., relative to basal promoter activity. Significant increases in promoter activity are indicated [**P<0.01 and ***P<0.001 (*n*=3)]. n.s., not significant.

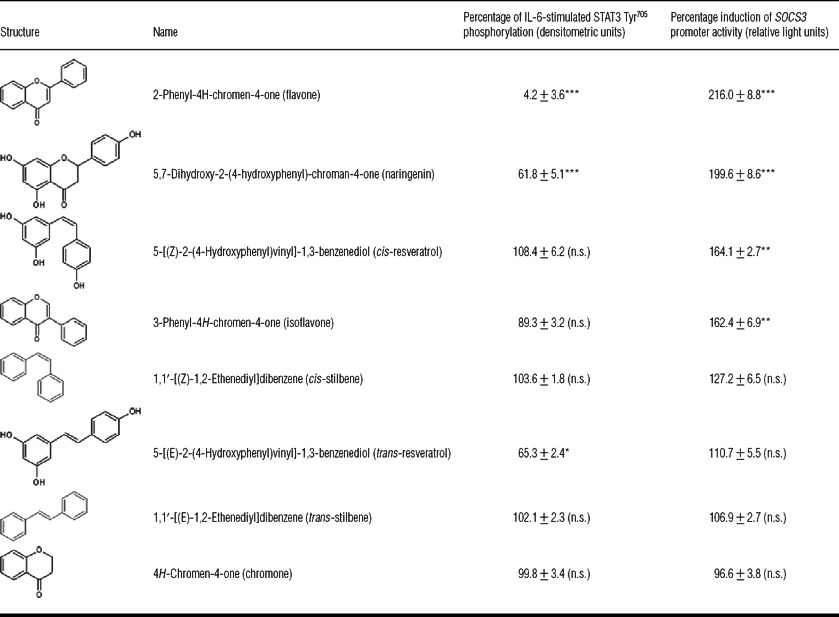

### Compound C antagonizes flavone-induced SOCS3 expression

We next sought to determine the molecular mechanisms by which flavone induces *SOCS3* gene expression in HUVECs and first investigated the role of AMPK (AMP-activated protein kinase), since previous work implicates a role for flavanoids in modulating AMPK activity [27]. To this end we used compound C, which is generally marketed as an inhibitor of AMPK; however, a number of off-target effects have been reported in the literature [28]. Intriguingly we found that 10 μM compound C effectively inhibited flavone-induced SOCS3 expression, but not inhibition of IL-6-promoted STAT3 activation in HUVECs (Figure 4a). This suggests that compound C inhibits downstream signalling from flavone to *SOCS3* gene induction. Stimulation of HUVECs with flavone was found to elicit a degree of AMPK activation, as determined by immunoblotting cell extracts with phospho-specific antibodies that detect the AMPK substrate ACC (Ser^79^); however, direct activation of AMPK with the specific AMPK agonist 10 μM A769622 failed to induce SOCS3 expression (Figure 4a). In addition, flavone-activated AMPK does not appear to be involved in the inhibition of STAT3 activation, since compound C treatment blocked ACC phosphorylation in response to flavone, but had little effect on flavone-inhibited STAT3 phosphorylation (Figure 4a). These results suggest that although flavone can activate AMPK in HUVECs, this activity is not linked to the induction of the *SOCS3* gene or inhibition of STAT3 activation. However, given that compound C effectively inhibits flavone action on the *SOCS3* gene, we decided to use compound C as a chemical probe to identify signalling pathways that may couple flavone stimulation to SOCS3 induction.

To this end we tested the ability of compound C to inhibit SOCS3 induction promoted by activation of the PKC (protein kinase C) pathway, the JAK/STAT3 signalling pathway and the cAMP cascade, since all of these pathways have previously been implicated in controlling *SOCS3* gene induction in HUVECs [10,21,24,29]. We first activated PKC signalling in HUVECs by stimulating cells with the phorbol ester PMA (Figure 4b). PMA (100 nM) treatment produced a robust 4-fold increase in SOCS3 protein expression, which was significantly inhibited by the general PKC inhibitor 10 μM Gö6983, but not by 10 μM compound C (Figure 4b). The fact that PMA-induced SOCS3 expression is inhibited by Gö6983 but not compound C suggests that PKC activation leads to SOCS3 induction through a pathway distinct from that regulated by flavone. Similarly, activation of JAK/STAT3 signalling following IL-6 treatment of HUVECs also led to a significant 8-fold induction in SOCS3 protein expression (Figure 4b), which was effectively suppressed by co-incubation with the JAK inhibitor 10 μM INCB018429, but not 10 μM compound C (Figure 4b). Again this suggests that IL-6-mediated induction of SOCS3 expression is dependent on JAK activity; however, the actions of flavone on SOCS3 expression must occur independently of JAK/STAT3 signalling since compound C treatment had little effect on IL-6 signalling. To test the role of cAMP in mediating SOCS3 induction, HUVECs were stimulated with a combination of 10 μM forskolin and 10 μM rolipram to elevate intracellular cAMP levels (Figure 4b). cAMP has previously been shown to be a potent inducer of SOCS3 expression in HUVECs through the combined action of the ERK and EPAC1 signalling pathways [29,30]. Accordingly, elevations in intracellular cAMP levels following forskolin/rolipram treatment provoked a significant 6-fold increase in SOCS3 protein expression (Figure 4b), which was significantly inhibited (by approximately 30%, *P*<0.05) by both 10 μM compound C and the inhibitor of ERK activation 1 μM PD184352 [31]. That cAMP-induced SOCS3 induction was inhibited by both compound C and an ERK inhibitor indicates that flavone may use similar signalling mechanisms to those used by cAMP to induce *SOCS3* gene expression. We therefore investigated the roles of cAMP and ERK signalling in mediating the actions of flavone on *SOCS3* gene induction.

### *SOCS3* gene induction by flavone is dependent on ERK activity

We have previously demonstrated that induction of *SOCS3* gene expression by cAMP is dependent on the combined actions of cAMP on EPAC1 activation and ERK signalling in HUVECs [29,32]. We therefore investigated the involvement of these two pathways in mediating induction of SOCS3 by flavone. Given that flavanoids inhibit CK2 (casein kinase 2) [33], an enzyme known to antagonize cAMP production [34], and that resveratrol has recently been described as an inhibitor of PDE4 activity [35], we decided to test the involvement of cAMP in flavone-induced SOCS3 induction and inhibition of STAT3 activation. We found that flavone, resveratrol and rolipram were all able to enhance the ability of the cAMP-elevating agent forskolin to inhibit IL-6-promoted STAT3 activation (Supplementary Figure S3a at http://www.biochemj.org/bj/454/bj4540283add.htm), indicating that both flavone and resveratrol could potentially enhance cAMP production either through inhibition of CK2 or PDE4. However, direct measurement of cAMP levels following treatment of HUVECs with combinations of flavone, resveratrol, rolipram and forksolin demonstrated that rolipram, and to a minor extent resveratrol, but not flavone, were able to potentiate forskolin-induced cAMP synthesis in HUVECs (Supplementary Figure S3b), indicating that flavone induces *SOCS3* gene induction independently of cAMP. To determine whether the combined actions of flavanoid treatment and IL-6 stimulation effected cAMP levels, we examined whether co-stimulation of HUVECs with IL-6 and forskolin, and either flavone, reseveratrol or rolipram, effects phosphorylation of the PKA (protein kinase A) substrate VASP on Ser^157^ (Supplementary Figure S3c). Results demonstrated that IL-6 did not promote VASP Ser^157^ phosphorylation, after either 30 min or 5 h stimulation (Supplementary Figure S3c). In contrast, a combination of forskolin and IL-6 promoted a robust phosphorylation of VASP after 30 min stimulation, which was enhanced by 5 h co-treatment with rolipram and, to a lesser extent, resveratrol, but not by flavone (Supplementary Figure S3c). Taken together these results indicate that the ability of flavone to induce SOCS3 expression and inhibit IL-6 promoted STAT3 activation occurs independently of elevations in intracellular cAMP in HUVECs.

**Figure 5:**
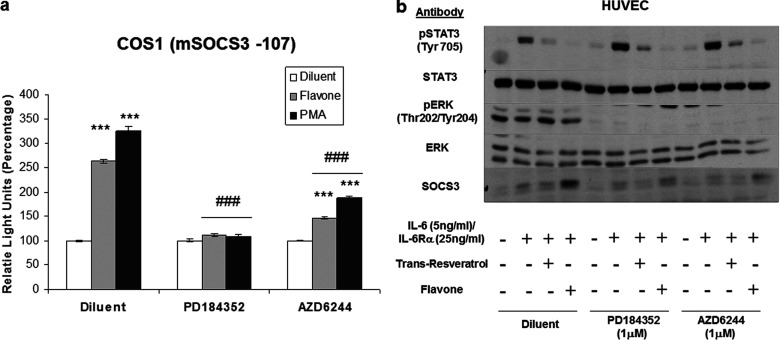
ERK MAPK is required for flavone induced *SOCS3* expression, but not for inhibition of STAT3 phosphorylation (**a**) COS-1 cells were transfected with a luciferase reporter construct encoding the minimal murine *Socs3* promoter [24]. In addition, cells were co-transfected with *Renilla* luciferase vector to normalize luciferase activity and to correct for transfection efficiency. Cells were then stimulated overnight with either 100 μM flavone or 100 nM PMA, in the presence of 1 μM of the MEK inhibitors PD184352 or AZD6244. Cell extracts were prepared and luciferase activities from three separate experiments were quantified. Significant increases in luciferase activity relative to diluent-treated cells are indicated [***P<0.001 (*n*=3)]. Significant reductions in luciferase activity relative to stimulated cells are also indicated (###*P*<0.001). (**b**) HUVECs pre-incubated with either PD184352 or AZD6244 and then stimulated with IL-6 (5 ng/ml) plus IL-6Rα (25 ng/ml), flavone or *trans*-resveratrol. Cell extracts were then immunoblotted with antibodies against SOCS3, and total and phosphorylated forms of ERK and STAT3 as indicated.

The results shown in Figure 4(b) indicate that induction of SOCS3 expression by flavone may share downstream signalling with the cAMP signalling cascade. Since flavone does not appear to elevate intracellular cAMP levels itself in HUVECs (Supplementary Figure S3) then it could possibly be interacting with downstream signalling elements. In this regard we have previously shown that elevations in intracellular cAMP activates the MAPK (mitogen-activated protein kinase) ERK in HUVECs, and this activation appears to be required for cAMP to induce *SOCS3* gene expression [24,32]. We therefore investigated whether ERK activation is also a prerequisite for flavone-induced SOCS3 expression. We therefore examined whether two inhibitors of ERK activation, the MEK (MAPK/ERK kinase) inhibitors PD184352 and AZD6244, could antagonize the actions of flavone on SOCS3 induction and inhibition of STAT3 activation. We found that both inhibitors effectively blocked the ability of flavone to induce *SOCS3* promoter activity in transfected COS-1 cells and SOCS3 protein synthesis in HUVECs (Figures 5a and 5b). We also noted that, although both ERK inhibitors effectively blocked flavone-induced SOCS3 production in HUVECs, flavone did not produce an appreciable activation of ERK (Figure 5b), indicating that it is basal, rather than stimulated, levels of ERK that are required for *SOCS3* gene induction by flavone in these cells.

**Figure 6:**
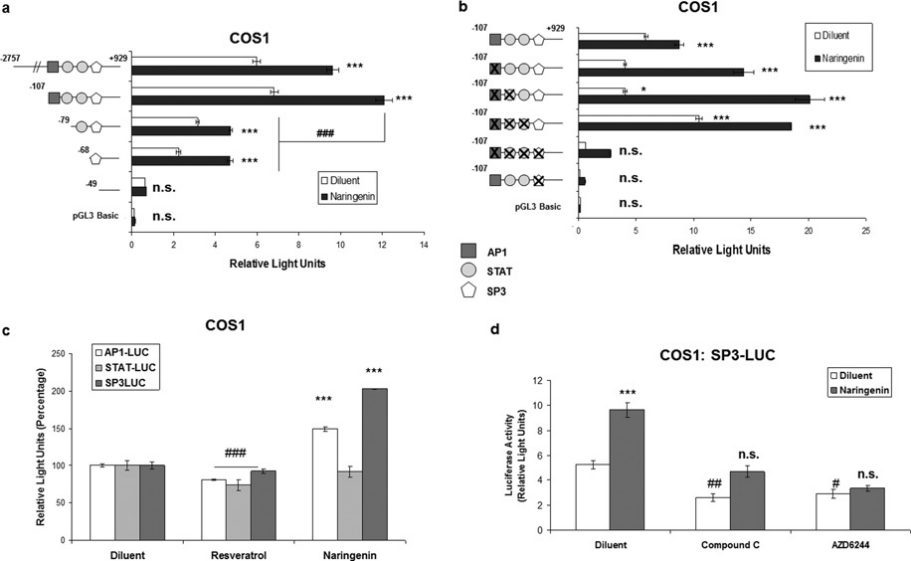
*SOCS3* gene induction by naringenin is dependent on SP3 transcription factors (**a**) COS-1 cells were transfected with firefly luciferase reporter constructs containing *Renilla* luciferase and truncates of the murine *Socs3* promoter as indicated [24]. Cells were then stimulated for 16 h with 100 μM naringenin, after which time cells were harvested and luciferase activities determined. The relative positions of putative transcription factor-binding sites within the minimal promoter region are shown in the schematic diagram on the left-hand side of the histogram. Results are means±S.E.M. of absolute relative light units (RLUs) from three separate experiments and significant differences relative to cells stimulated with diluent alone are indicated (***P<0.001). Significant reductions in naringenin-promoted luciferase activities relative to stimulated levels obtained from the minimal *Socs3* promoter construct are also indicated (###*P*<0.0001). (**b**) COS-1 cells were transfected with vectors encoding *Renilla* luciferase, together with luciferase vectors encoding the minmal murine *Socs3* minimal promoter (mSOCS3-107) in which individual transcription factor-binding sites had been deleted. Cells were then stimulated for 16 h with 100 μM naringenin, after which time cells were harvested and luciferase activities determined. Results are means±S.E.M. of absolute RLUs from three separate experiments. Significant differences relative to cells transfected with non-mutated promoter and stimulated with diluent alone are indicated (*P<0.05, **P<0.01 and ***P<0.001). (**c**) COS-1 cells were transfected with luciferease reporter constructs encoding multiple consensus DNA-binding sites for STAT, AP1 or SP3 transcription factors. Cells were then stimulated for 16 h with 100 μM naringenin or *trans*-resveratrol. Luciferase activities were determined and significant increases or decreases in luciferase activity relative to diluent-treated cells are indicated [***P<0.001 and ###*P*<0.001 respectively (*n*=3)]. (**d**) COS-1 cells were transfected with luciferease reporter constructs encoding multiple consensus DNA-binding sites for SP3 transcription factors. Cells were then stimulated for 16 h with 100 μM naringenin in the presence or absence of either 10 μM compound C or the MEK inhibitor 10 μM AZD6244. Luciferase activities were determined (*n*=3) and significant increases (***P<0.001) or decreases (#*P*<0.05 and ##*P*<0.01) in luciferase activity relative to diluent-treated cells are indicated. n.s., not significant.

We also noted that neither PD184352 nor AZD6244 affected the ability of resveratrol and flavone to block IL-6-promoted STAT3 activation (Figure 5b), indicating that the actions of these compounds on STAT3 activation occur independently of ERK and SOCS3 induction. This is borne out by the observation that naringenin treatment of HUVECs failed to affect IL-6-promoted phosphorylation of the STAT3 ERK-target site, Ser^727^ (Supplementary Figure S1b). Indeed, IL-6-promoted phosphorylation of STAT3 on Tyr^705^ appears to be enhanced, rather than inhibited, in the presence of PD184352 and AZD6244 (Figure 5b). These results reinforce the idea that transcription factors other than STAT3 are responsible for *SOCS3* gene induction in response to flavone treatment. Indeed, our previous work has demonstrated that the activity of the minimal *SOCS3* promoter is dependent on basal activity of ERK and the ERK-targeted transcription factors AP1 and SP3, in addition to STAT3 [24]. We therefore examined the role of these three transcription factors in supporting the activity of the *SOCS3* promoter in luciferase reporter assays as described previously [21,24]. We first transfected COS-1 cells with luciferase reporter constructs containing various lengths of the murine *Socs3* promoter spanning −49 to −2757 nt from the initiating ATG (Figure 6a). We found that naringenin treatment led to a significant approximate 1.5-fold induction of the full-length −2757 promoter and the −107 minimal promoter (Figure 6a). Further promoter truncation, removing the AP1- and STAT-binding sites, was associated with a reduction in basal, but not naringenin-stimulated, activity (Figure 6a). However, further truncation, leading to the loss of the SP3-binding site, led to a loss of both basal and naringenin-stimulated promoter activity (Figure 6a), suggesting that SP3 transcription factors may be important for *SOCS3* gene induction by flavanoids. This idea is supported by further experiments where we ablated individual transcription factor-binding sites within the *SOCS3* minimal promoter (Figure 6b). We found that sequential deletion of the AP1- and STAT-binding sites had little effect on naringenin-promoted *SOCS3* promoter activity (Figure 6b). However, ablation of the SP3-binding site, by itself or in combination with the AP1- and STAT-binding sites, led to a dramatic reduction in both basal and naringenin-stimulated promoter activity (Figure 6b), indicating a potential role for SP3 transcription factors in the control of *SOCS3* gene activity. This idea is supported by observations that treatment of COS-1 cells with naringenin led to an increase in both AP1 and SP3 transcription factor activity (Figure 6c), as determined by the use of luciferase reporter constructs as described previously [24]. This is in contrast with *trans*-resveratrol, which generally suppressed AP1, STAT and SP3 transcription factor activity (Figure 6c). In addition, and consistent with the idea that basal ERK activity is required for regulation of *SOCS3* gene activity, the ERK inhibitor AZD6244 suppressed both basal and naringenin-stimulated SP3 transcription factor activity (Figure 6d). Moreover, basal and naringenin-stimulated SP3 activity were also inhibited by co-treatment with compound C, which supports the idea that SP3 transcription factors are involved in the regulation of *SOCS3* gene activity by flavanoids.

In summary, in the present paper we describe a new class of chemical probes based on the flavone core structure that have the novel combined actions of STAT3 inhibitor and inducer of *SOCS3* gene expression. The ability of these compounds to induce *SOCS3* gene expression appears to rely on ERK-dependent SP3 transcription factor activation (Figure 6). Our objective is to use these probes to identify their protein targets in VECs. It is highly likely that the cellular target of flavone will be a kinase or phosphatase, since flavanoids have been reported to act on protein kinases, including the serine/threonine kinases PKC, Aurora-A, -B and -C and AMPK, the tyrosine kinases JAK3, PIM1 and PDGFRα (platelet-derived growth factor receptor α) and PDGFRβα, and lipid kinases, including PI3K (phosphoinositide 3-kinase) [27,36–39]. Identifying the protein targets of flavone will serve as a starting point for the development of novel therapeutics to combat chronic inflammatory diseases where there is hyperactivation of JAK/STAT3 signalling. This approach could have advantages over existing anti-IL-6 therapies; for example, monoclonal antibodies against the IL-6R, e.g. tocilizumab [40], are in regular clinical use; however, humanized antibodies are expensive to produce and store, have a limited shelf life and require regular injections for effective treatment. Current therapeutic small-molecule JAK/STAT3 inhibitors, such as tasocitinib [41], are cheap, easy to produce and can be taken orally, but effectively suppress SOCS3 activity [42] (Figure 3c). Since we have yet to identify the full range of proteins targeted by SOCS3 for proteosomal degradation [43], traditional drugs, such as tasocitinib, may have limited anti-inflammatory actions or unforeseen side effects when compared with the flavanoid-based STAT3 inhibitors/SOCS3-elevating agents described in the present paper [17]. In the experiments we carried out we used both naringenin and flavone at concentrations up to 100 μM because naringenin has been shown not to be cytotoxic to HUVECs at this concentration, and is in fact anti-apoptotic [44]. High levels of narignenin, between 200 and 400 μM, have been shown to be cytotoxic to cancer cells [45]; however, in general, flavanoids are not thought to be toxic and are readily bioavailable in foods; for example ingestion of 8 ml/kg grapefruit juice will provide a plasma concentration of naringenin of approximately 6 μM [46]. In the case of flavone, *in vivo* measurements have demonstrated that flavone acetate exerts anti-neoplastic actions at a plasma concentration of around 360 μM and is acutely lethal at 2.1 mM [47]. Plasma concentrations of flavone acetate between 360 μM and 2.1 mM induce delayed death [47]. Thus the actions of the flavanoids tested in the present study occur within the tolerated concentration range of these compounds and should therefore be useful to the wider scientific community for further functional testing.

## Online data

Supplementary data
