# Supplementary material for: Flavanoids induce expression of the suppressor of cytokine signalling 3 (SOCS3) gene and suppress IL-6-activated signal transducer and activator of transcription 3 (STAT3) activation in vascular endothelial cells
Source: Biochem J. 2013 Aug 9;454(Pt 2):283–93. doi: 10.1042/BJ20130481 (PMC3749869; doi:10.1042/BJ20130481)
Supplement: Supplementary data [file bj4540283add.pdf]

BJ Signal

Biochem. J. (2013) 454, 283–293 (Printed in Great Britain) doi:10.1042/BJ20130481

online data

SUPPLEMENTARY ONLINE DATA

Flavanoids induce expression of the suppressor of cytokine signalling 3 (*SOCS3*) gene and suppress IL-6-activated signal transducer and activator of transcription 3 (STAT3) activation in vascular endothelial cells

Jolanta WIEJAK*[1], Julia DUNLOP*[1], Simon P. MACKAY† and Stephen J. YARWOOD*[2]

*Institute of Molecular, Cell and Systems Biology, College of Medical, Veterinary and Life Sciences, University of Glasgow, Glasgow G12 8QQ, Scotland, U.K., and †Strathclyde Institute of Pharmacy and Biomedical Sciences, 161 Cathedral Street, University of Strathclyde, Glasgow G4 0RE, Scotland, U.K.

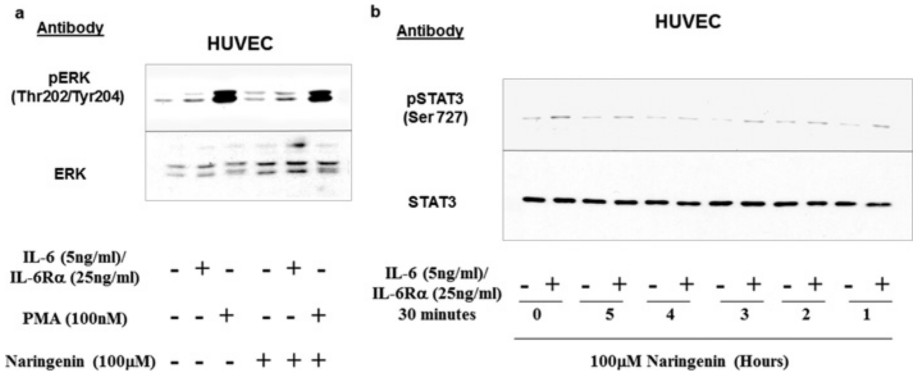

Figure S1 Naringenin has little effect on the phosphorylation of either ERK or STAT Ser^{727} in HUVECs

(**a**) HUVECs were pre-incubated for 5 h in the presence or absence of 100 μM naringenin. Cells were then stimulated with either diluent, IL-6 (5 ng/ml)/IL-6Rα (25 ng/ml) or the PKC activator 100 nM PMA. Cell extracts were then prepared and immunoblotted with anti-phospho-ERK or anti-total ERK antibodies as indicated. (**b**) HUVECs were pre-incubated for the times indicated with 100 μM naringenin and then stimulated for 30 min in the presence or absence of IL-6 (5 ng/ml)/IL-6Rα (25 ng/ml). Cell extracts were then immunoblotted with anti-phospho-STAT3 (Ser^{727}) or anti-total STAT3 antibodies as indicated.

[1] These authors contributed equally to this work.
[2] To whom correspondence should be addressed (email stephen.yarwood@glasgow.ac.uk).

© 2013 The Author(s)
The author(s) has paid for this article to be freely available under the terms of the Creative Commons Attribution Licence (CC-BY) (http://creativecommons.org/licenses/by/3.0/) which permits unrestricted use, distribution and reproduction in any medium, provided the original work is properly cited.

Biochemical Journal www.biochemj.org

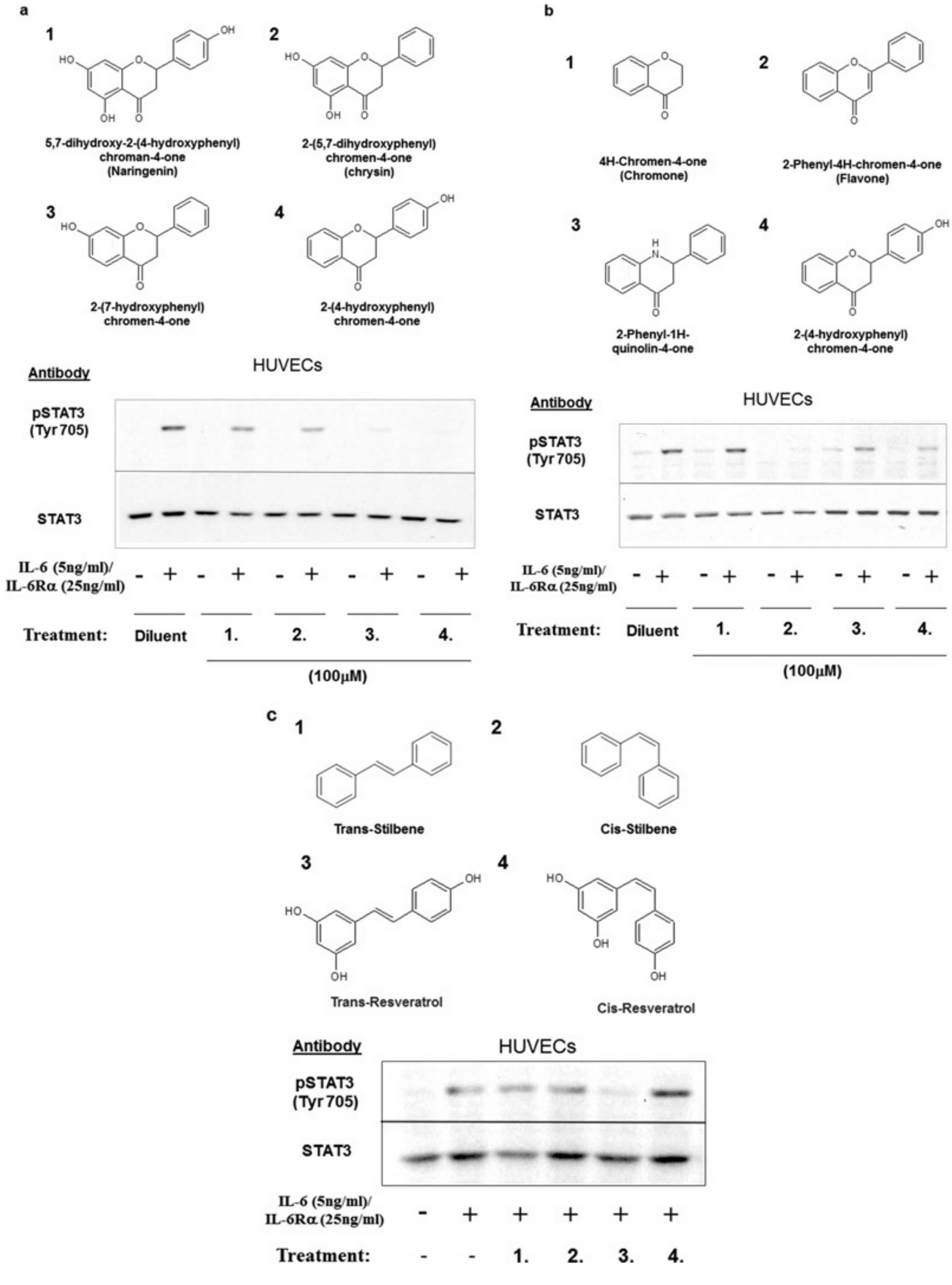

Figure S2 Effects of variations in flavanoid structure on the inhibition of IL-6-promoted STAT3 activation in HUVECs

(**a**) HUVECs were stimulated for 5 h with IL-6 (5 ng/ml) plus IL-6Rα (25 ng/ml) in the presence or absence of either 100 μM naringenin, 2-(5,7-dihydroxyphenyl) chromen-4-one, 2-(7-hydroxyphenyl) chromen-4-one or 2-(4-hydroxyphenyl) chromen-4-one (structures are shown in the top panel). Cell lysates were then prepared and immunoblotted with anti-STAT3 and anti-phospho-STAT3 (Tyr^{705}) antibodies as shown in the bottom panel. Results indicate that 2-(7-hydroxyphenyl) chromen-4-one and 2-(4-hydroxyphenyl) chromen-4-one are the most effective inhibitors of IL-6-stimulated STAT3 phosphorylation in HUVECs. (**b**) HUVECs were stimulated for 5 h with IL-6 (5 ng/ml) plus IL-6Rα (25 ng/ml) in the presence or absence of either 100 μM 4H-chromen-4-one, 2-phenyl-4H-chromen-4-one, 2-phenyl-4H-chromen-4-one, 2-phenyl-1H-quinolin-4-one or 2-(4-hydroxyphenyl) chromen-4-one (structures are shown in the top panel). Cell lysates were then prepared and immunoblotted with anti-STAT3 and anti-phospho-STAT3 (Tyr^{705}) antibodies as shown in the bottom panel. Results indicate that 2-phenyl-1H-quinolin-4-one and 2-(4-hydroxyphenyl) chromen-4-one are the most effective inhibitors of IL-6-stimulated STAT3 phosphorylation in HUVECs. (**c**) HUVECs were stimulated for 5 h with IL-6 (5 ng/ml) plus IL-6Rα (25 ng/ml) in the presence or absence of either 100 μM *trans*-stilbene, *cis*-stilbene, *trans*-resveratrol or *cis*-resveratrol (structures are shown in the top panel). Cell lysates were then prepared and immunoblotted with anti-STAT3 and anti-phospho-STAT3 (Tyr^{705}) antibodies as shown in the bottom panel. Results indicate that *trans*-resveratrol is the most effective inhibitor of IL-6-stimulated STAT3 phosphorylation in HUVECs.

© 2013 The Author(s)
The author(s) has paid for this article to be freely available under the terms of the Creative Commons Attribution Licence (CC-BY) (http://creativecommons.org/licenses/by/3.0/) which permits unrestricted use, distribution and reproduction in any medium, provided the original work is properly cited.

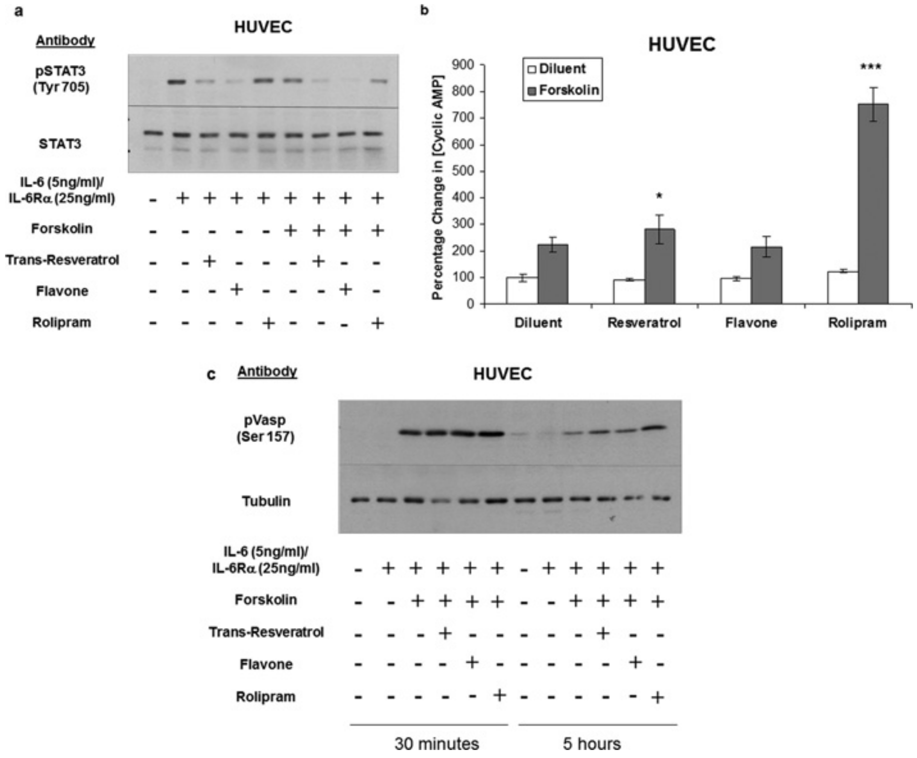

Figure S3 Flavone-induced SOCS3 expression and inhibition of STAT3 is independent of increases in intracellular cAMP levels

(**a**) HUVECs were stimulated for 5 h with the indicated combinations of IL-6 (5 ng/ml)/IL-6Rα (25 ng/ml), forskolin (10 μM), flavone (100 μM), *trans*-resveratrol (100 μM) and rolipram (10 μM). Cell extracts were then immunoblotted with anti-SOCS3, anti-STAT3 and anti-phospho-STAT3 antibodies as indicated. (**b**) HUVECs were stimulated for 5 h with forskolin (10 μM) in the presence or absence of flavone (100 μM), *trans*-resveratrol (100 μM) or rolipram (10 μM) as indicated. cAMP levels in cell extracts were then quantified using a luminescent ELISA-based assay, as described in the Materials and methods of the main paper. Results are the average of an experiment carried out on three separate occasions and significant increases in cAMP levels with respect to diluent-treated cells are indicated ($^*P < 0.05$ and $^{***}P < 0.001$). (**c**) HUVECs were stimulated for either 30 min or 5 h in the presence or absence of IL-6 (5 ng/ml)/IL-6Rα (25 ng/ml) together with the indicated combinations of forskolin, flavone, reseveratrol and rolipram. Cell extracts were then prepared and immunoblotted with anti-phospho-VASP (Ser^{157}) and anti-tubulin antibodies.

Received 4 April 2013/12 June 2013; accepted 19 June 2013
Published as BJ Immediate Publication 19 June 2013, doi:10.1042/BJ20130481

© 2013 The Author(s)
The author(s) has paid for this article to be freely available under the terms of the Creative Commons Attribution Licence (CC-BY) (http://creativecommons.org/licenses/by/3.0/) which permits unrestricted use, distribution and reproduction in any medium, provided the original work is properly cited.
